# Antioxidant Strategies to Modulate NETosis and the Release of Neutrophil Extracellular Traps during Chronic Inflammation

**DOI:** 10.3390/antiox12020478

**Published:** 2023-02-14

**Authors:** Line A. E. Hallberg, Kristine Barlous, Clare L. Hawkins

**Affiliations:** Department of Biomedical Sciences, University of Copenhagen, DK-2200 Copenhagen, Denmark

**Keywords:** neutrophil, neutrophil extracellular trap, hypochlorous acid, myeloperoxidase, chronic inflammation, thiocyanate, selenocyanate, TEMPO

## Abstract

Extracellular traps are released by neutrophils and other immune cells as part of the innate immune response to combat pathogens. Neutrophil extracellular traps (NETs) consist of a mesh of DNA and histone proteins decorated with various anti-microbial granule proteins, such as elastase and myeloperoxidase (MPO). In addition to their role in innate immunity, NETs are also strongly linked with numerous pathological conditions, including atherosclerosis, sepsis and COVID-19. This has led to significant interest in developing strategies to inhibit NET release. In this study, we have examined the efficacy of different antioxidant approaches to selectively modulate the inflammatory release of NETs. PLB-985 neutrophil-like cells were shown to release NETs on exposure to phorbol myristate acetate (PMA), hypochlorous acid or nigericin, a bacterial peptide derived from *Streptomyces hygroscopicus*. Studies with the probe R19-S indicated that treatment of the PLB-985 cells with PMA, but not nigericin, resulted in the production of HOCl. Therefore, studies were extended to examine the efficacy of a range of antioxidant compounds that modulate HOCl production by MPO to prevent NETosis. It was shown that thiocyanate, selenocyanate and various nitroxides could prevent NETosis in PLB-985 neutrophils exposed to PMA and HOCl, but not nigericin. These results were confirmed in analogous experiments with freshly isolated primary human neutrophils. Taken together, these data provide new information regarding the utility of supplementation with MPO inhibitors and/or HOCl scavengers to prevent NET release, which could be important to more specifically target pathological NETosis in vivo.

## 1. Introduction

Neutrophils are the most abundant granulocyte in the circulation and a key component of the innate immune response [1,2]. Neutrophils combat pathogens by different mechanisms, including phagocytosis, degranulation and the release of neutrophil extracellular traps (NETs) [1]. NETs were first characterized in 2004, when Brinkmann and colleagues demonstrated that neutrophils could expel spindles of decondensed DNA together with histones, myeloperoxidase (MPO), elastase and other granule proteins [3]. An increasing number of extrinsic and intrinsic stimuli can trigger NET release, which leads to DNA release from the nuclei and/or mitochondria into the extracellular space [4,5,6]. These stimuli include various pathogens and pro-inflammatory species such as cytokines, chemokines and the MPO-derived oxidant, hypochlorous acid (HOCl) [6,7,8,9]. Phorbol 12-myristate 13-acetate (PMA) has been used extensively as an inducer of NETosis in in vitro and ex vivo experiments with neutrophils and other types of immune cells (e.g., [5,8,10,11]). It is a protein kinase C (PKC) activator, which promotes neutrophil activation and assembly of the NADPH oxidase complex resulting in the production of superoxide (O_2_^∙−^), which is involved, together with MPO, in driving NET release [5,8,11,12]. This has been suggested to involve the generation of HOCl, which is able to independently induce extracellular trap release in neutrophils [7,13] and other cells, including macrophages [14].

Two forms of NETosis have been proposed, which involve either a slow lytic (suicidal) or rapid non-lytic (vital) pathway [6,15]. Both suicidal and vital forms of NETosis are associated with the activation of NADPH oxidase and the generation of reactive oxygen species (ROS), including HOCl [6,12,16]. Although there is a clear association between the generation of ROS with NET release, ROS generation and NET release can also occur independently of NADPH oxidase by reactions involving mitochondria [4,16]. Regardless of the mechanism of NETosis, NETs constitute an important innate immune defence, particularly against large pathogens that are difficult to phagocytose [17]. The DNA backbone, together with the presence of histones, MPO and other granule proteins, can all contribute to pathogen killing in the extracellular environment and assist in clearing infection [18,19]. However, despite their importance in the innate immune response, there is increasing evidence that NETs can also promote inflammation, thrombosis and damage to host tissue, culminating in organ failure and the propagation of disease [6,20].

There is clear evidence for the role of NETs in the development of a range of acute and chronic diseases, including COVID-19 [21], sepsis [22], cancer [23], autoimmune and renal disease [24], diabetes mellitus [25] and cancer progression and metastasis [26]. NETs have also emerged as key contributors to the development of cardiovascular disease [27], and the vascular complications of other pathologies, such as diabetes mellitus [25,28], systemic lupus erythematosus [29] and COVID-19 [21]. As a result, there is significant interest in therapeutically targeting NETs whilst minimizing the risk of complications resulting from a compromised ability to clear infections [30,31]. A growing number of FDA-approved drugs are known to exert NET-directed effects via different pathways, including by modulating the inflammatory environment, blocking interactions with platelets, inhibiting elastase or neutralizing the effects of extracellular histones [31].

NET release can be modulated by limiting the production of ROS and HOCl by inhibiting NADPH oxidase and/or MPO or by supplementing with an antioxidant. For example, metformin [32] and diphenyleneiodonium (DPI) [33] can decrease NETosis by their ability to inhibit NADPH oxidase and/or the production of mitochondrial ROS. Similarly, supplementation with different ROS-scavenging compounds, including N-acetylcysteine [34], resveratrol [35], selenomethionine [36] and flavonoids [37] can also be effective in decreasing the release of NETs. However, achieving a sufficiently high concentration of the scavenger compound to attain efficacy in vivo can be challenging.

In this study, we examined the ability of different antioxidants, including thiocyanate (SCN^−^), selenocyanate (SeCN^−^), TEMPO and 4-amino-TEMPO to modulate NET release. These compounds are expected to influence NETosis by a combination of mechanisms, including inhibition of O_2_^∙−^ by NADPH oxidase, inhibition of HOCl production by MPO and/or direct reaction with HOCl [38,39], and therefore may exert a more potent modulatory effect on ROS-dependent NETosis. Experiments were performed with the neutrophil PLB-985 cell line differentiated with all-trans retinoic acid (ATRA) to mimic granulopoiesis [40] and freshly isolated human neutrophils. In each case, the neutrophils were stimulated with the NET inducers PMA, HOCl or nigericin, a bacterial peptide derived from *Streptomyces hygroscopicus* [11] in the absence or presence of each antioxidant, which could be relevant for the development of new approaches to target NETosis more selectively in disease.

## 2. Materials and Methods

### 2.1. Reagents and Materials

All aqueous solutions were prepared using nanopure H_2_O from a Milli-Q system (Millipore). All chemicals and reagents were of the highest purity available and purchased from Sigma-Aldrich/Merck (Søborg, Denmark) unless stated otherwise. The concentration of HOCl was determined by UV absorbance at 292 nm at pH 11 using an extinction coefficient of 350 M^−1^ cm^−1^ [41].

### 2.2. Culturing and Differentiation of PLB-985 Cells 

PLB-985 cells (human acute myeloid leukaemia cells, DSMZ, ACC 139) were maintained in an incubator at 37 °C + 5% CO_2_ and cultured in Roswell Park Memorial Institute 1640 medium (RPMI-1640) supplemented with 10% foetal bovine serum (FBS, Thermo Fisher, Waltham, MA, USA) and 1% penicillin/streptomycin (100 U/mL). The cells were passaged every 2–3 days and maintained at a density of 0.2 × 10^6^–1.5 × 10^6^ cells/mL. To induce differentiation of the PLB-985 cells, cells were seeded at a density of 0.3 × 10^6^ cells/mL in culture medium (4 volumes new RPMI-1640 media:1 volume cell conditioned media) supplemented with ATRA (2 µM) and dimethyl sulfoxide (DMSO, 1.3% *v/v*) for 72 h [40].

### 2.3. May–Grünwald–Giemsa (MGG) Staining

Differentiated PLB-985 cells (2 × 10^5^) were suspended in 1 mL of RPMI-1640 medium diluted 1:1 with PBS. The slides were prepared by injecting cell suspensions into a cytofunnel, which was inserted into a cytocentrifuge (Shandon, Thermo Fisher, 400 rpm, 10 min) and dried for 30 min. The samples on the slides were fixed in methanol for 5 min and stained using May–Grünwald eosin methylene blue solution (Merck) for 10 min followed by incubation in diluted Giemsa’s azur eosin methylene blue solution (Merck) for 15 min. Slides were washed in pH 6.5 PBS for 5 min and nanopure H_2_O for 1 min before drying for 60 min, and analysed on a BX51 microscope with a DP70 camera (Olympus, Ballerup, Denmark).

### 2.4. Analysis of Differentiated PLB-985 Cells by Flow Cytometry

PLB-985 cells (4 × 10^6^) (with and without differentiation) were isolated by centrifugation (300× *g*, 5 min) and resuspended in PBS supplemented with 0.5% (*w/v*) bovine serum albumin (BSA) and 2 mM EDTA before incubation with either IgG antibodies (1:100) and CD11b PE antibodies (1:100) (BD Biosciences, Lyngby, Denmark) for 15 min in the dark. Separate aliquots of cells were washed in PBS supplemented with 0.5% BSA and 2 mM EDTA, resuspended in PBS and either incubated with 7-aminoactinomycin D (7-AAD, Molecular Probes™, Thermo Fisher) for 15 min or left unstained. After washing and resuspension in PBS, cells were analysed by flow cytometry (Accuri C6 flow cytometer, BD Biosciences). Dead cells and cell debris were excluded using the 7-AAD stain.

### 2.5. Isolation of Primary Neutrophils from Human Buffy Coat Preparations

Freshly isolated buffy coats from anonymous donors were obtained, on the same day as the experiments, from a blood bank (Rigshospitalet, Copenhagen, Denmark). The buffy coat preparation was diluted 1:4 with warm Hank’s balanced salt solution (HBSS, Gibco, Thermo Fisher) at 37 °C before being carefully laid on top of Ficoll-Paque^TM^ PLUS 1:1.3 (Cytvia, Vallensbæk Strand, Denmark). The cells (granulocytes/erythrocytes) were isolated by centrifugation (400× *g*, 30 min) and suspended in 3 volumes of PBS and 1 volume of 4% (*w*/*v*) dextran with incubation for 30 min at 21 °C to remove red blood cells. The supernatant was collected and centrifuged (250× *g*, 5 min) and the cell pellet containing the neutrophils was suspended in 5 mL of red cell lysis buffer (Roche, Cat. No. 11,814 389 001, from Merck) for 10 min. The neutrophils were isolated by centrifugation (250× *g*, 5 min) and washed in warm (37 °C) HBSS until the supernatant was colourless, before suspending in HBSS for experiments.

### 2.6. Analysis of NET Release by Microscopy

Differentiated PLB-985 cells or primary neutrophils were carefully washed and resuspended in RPMI-1640 media before plating on chamber slides pre-coated with poly-L-lysine. After 1 h at 37 °C, the slides were washed with PBS and fresh RPMI-1640 media was added. The cells were treated with PMA (50 or 200 nM), HOCl (0.75 mM) or nigericin (15 µM) for 4 h at 37 °C with 5% CO_2_, before fixing the cells with 4% formaldehyde for 30 min. The slides were stained with SYTOX green (2 µM, 10 min; Thermo Fisher) after washing with HBSS. For immunocytochemistry experiments, the slides were washed in PBS containing 0.5% Tween and incubated overnight in 5% BSA in PBST at 4 °C. The slides were incubated with primary antibodies: anti-histone citrulline H3 (1:100; ab5103, Abcam, Cambridge, UK), anti-MPO (1:100; ab25989, Abcam) or anti-elastase (1:100; ab21595, Abcam) for 2 h at 21 °C. The cells were washed in PBST and incubated with secondary antibodies (1:1000; AlexaFluor^®^ 647 and 1:1000 AlexaFluor^®^ 488, Abcam) for 2 h in the dark. Images were captured using a fluorescent microscope (Olympus, BX51; ZEISS widefield fluorescent microscope; ZEISS Axio Scan.Z1) and processed using ZEN Blue edition software.

### 2.7. Quantification of NETs by Fluorescence

The NETs were removed from the differentiated PLB-985 cells or primary neutrophils by the addition of DNase I (40 U) and incubation for 15 min at 37 °C and 5% CO_2_ before the addition of 5 mM EDTA (Thermo Fisher) to inactivate the DNase I. The cells and cellular debris were pelleted by centrifugation (300× *g* for 5 min), and the DNA present in the supernatants was quantified using the Quant-iT™ PicoGreen™ dsDNA assay kit according to the manufacturer’s instructions (Thermo Fisher) using black-well 96-well plates. The fluorescence was measured at λex 480 nm and λem 520 using a SpectraMaX^®^ i3x Multi-Mode Microplate Reader (Molecular Devices, Wokingham, UK).

### 2.8. Quantification of HOCl Production by PLB-985 Cells

Differentiated PLB-985 cells (2 × 10^6^ cells/mL) were incubated with the fluorescent HOCl probe R19-S (10 µM; Futurechem, Seoul, Republic of Korea) for 10 min before treatment with PMA (200–400 nM) or nigericin (10–20 µM). The fluorescence from the cells was measured over 4 h at λex 515 nm and λem 550 using a SpectraMaX^®^ i3x Multi-Mode Microplate Reader (Molecular Devices).

### 2.9. Statistical Analyses

Statistical analyses were performed using GraphPad Prism (version 9; GraphPad Software) using 1-way or 2-way ANOVA with *p* < 0.05 taken as significant. Data represent mean ± S.E.M. from at least 3 independent experiments in each case, with the details of the specific multiple comparison tests outlined in the figure captions.

## 3. Results

### 3.1. Differentiation of the PLB-985 Cell Line and Stimulation to Release NETs

**Figure 1:**
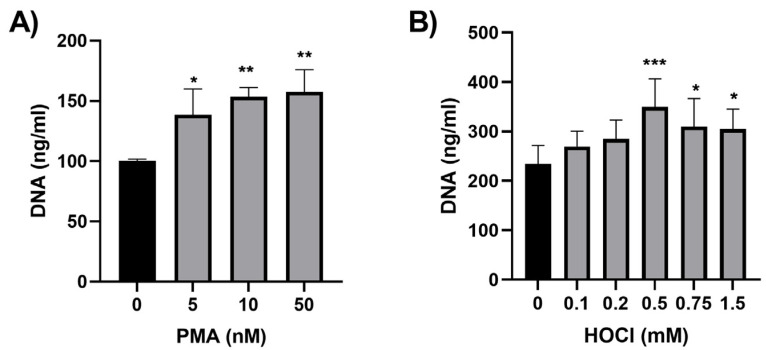
PLB-985 cells treated with PMA or HOCl release DNA into the extracellular space. Differentiated PLB-985 cells (1 × 10^6^ cells/mL) were treated with (**A**) PMA (0–50 nM) and (**B**) HOCl (0–1.5 mM) for 4 h before quantification of NET release by addition of DNase (40 U, 15 min) and determination of extracellular DNA using the Quant-iT^TM^ PicoGreen^TM^ dsDNA assay kit. Data are the mean ± S.E.M. of 3–6 experiments. *, ** and *** indicate a significant (*p* < 0.05, 0.01, 0.001) change on comparing the PMA- or HOCl-treated cells to the non-treated control by one-way ANOVA with Dunnett’s multiple comparisons test.

PLB-985 cells were differentiated into neutrophil-like cells using ATRA (2 µM) and DMSO (1.3% *v*/*v*) for 72 h to mimic granulopoiesis and maturation, as described previously [40]. This treatment resulted in significant morphological changes in comparison to non-differentiated cells with a marked change in the shape of the nuclei, which were initially large and oval becoming more segmented over 72 h, consistent with a mature neutrophil (Figure S1) [1]. Neutrophil differentiation was further validated by an increase in the expression of the maturation marker CD11b following 72 h treatment with ATRA and DMSO (Figure S2A,B) [42,43]. Initial studies were performed to examine the release of NETs from the differentiated PLB-985 cells following exposure to PMA or HOCl. A dose-dependent increase in the release of DNA from the cells was observed on exposure of the PLB-985 cells to PMA (5–50 nM) and HOCl (0.1–1.5 mM) for 4 h, as assessed by changes in fluorescence of the dsDNA stain, PicoGreen (Figure 1A,B).

**Figure 2:**
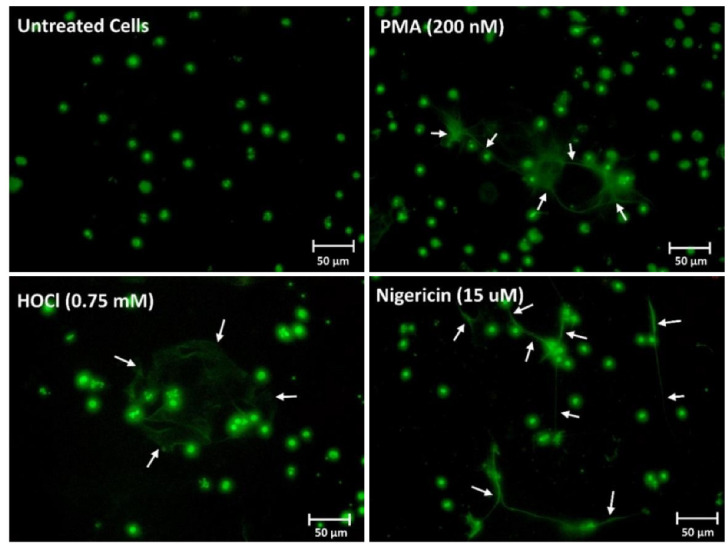
PMA, HOCl and nigericin induce NET release in differentiated PLB-985 cells. Differentiated PLB-985 cells (4 × 10^5^ cells) were treated with PMA (200 nM), HOCl (0.75 mM) or nigericin (15 µM) for 4 h at 37 °C. After the treatment, the cells were fixed in 4% formaldehyde for 30 min and the DNA was stained with SYTOX green (2 µM) for 10 min in the dark. The NETs are indicated by the white arrows. Images are representative of at least 3 independent experiments.

To confirm that the changes in fluorescence resulted from NET release, fluorescence microscopy was performed with extracellular DNA visualized by staining with SYTOX green. In this case, the differentiated PLB-985 cells were exposed to PMA (200 nM), HOCl (0.75 mM) or the bacterial peptide nigericin (15 μM) for 4 h (Figure 2). The presence of web-like structures of DNA extruding from the cells was observed with each treatment, but not in the non-treated control cells (Figure 2). Additional immunocytochemistry experiments were performed with PMA-treated cells to show that the extracellular DNA stained with SYTOX green colocalized with citrullinated histone H3, as a further marker of NET release (Figure S3) [44].

### 3.2. PLB-985 Cells Stimulated with PMA but Not Nigericin Produce HOCl

**Figure 3:**
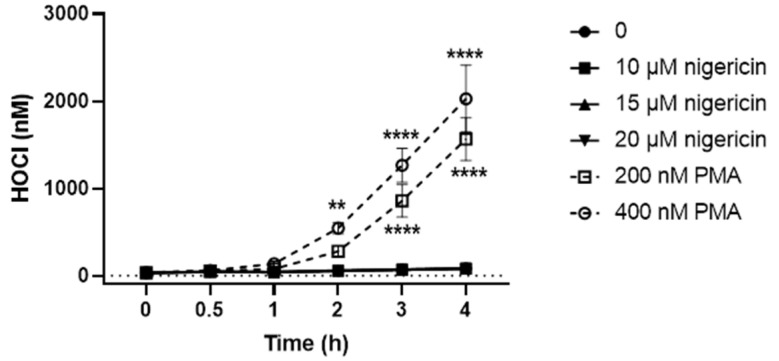
Differentiated PLB-985 cells produce HOCl when stimulated with PMA, but not with nigericin. Differentiated PLB-985 cells (2 × 10^6^ cells/mL) were stimulated with PMA (200 or 400 nM) or nigericin (10, 15 or 20 µM) in the presence of R19-S (10 µM), with changes in fluorescence recorded at **λ**_EX_ 515 nm and **λ**_EM_ 550 nm over 4 h. Symbols ●, ■, ▲ and ▼ are overlaid, as no HOCl was detectable on stimulation with nigericin. Data represent the mean ± S.E.M. of 3 experiments. **, and **** indicate a significant (*p* < 0.01, 0.0001) change on comparing each concentration of PMA to the non-treated control by a two-way ANOVA with Dunnett’s multiple comparisons test.

After confirming that the differentiated PLB-985 can be stimulated to release NETs on exposure to PMA and nigericin, experiments were then performed to examine the production of HOCl, which was shown to induce NET release. The differentiated PLB-985 cells were stimulated with PMA (200 and 400 nM) or nigericin (10, 15 and 20 µM) for 4 h in the presence of the HOCl-probe R19-S (10 µM) [45,46]. A time-dependent increase in R19 fluorescence was observed on exposure of the cells to PMA but not nigericin (Figure 3). With PMA, the concentration of HOCl produced was also dependent on the concentration of PMA, with higher amounts observed on treating the cells with 400 nM compared to 200 nM PMA (Figure 3).

### 3.3. Efficacy of Antioxidants in Modulating NET Release Observed on Stimulation of Neutrophils with PMA, HOCl or Nigericin

**Figure 4:**
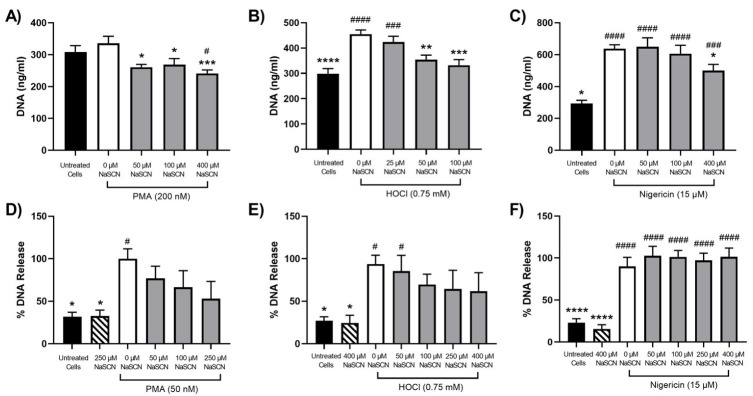
Effect of SCN^−^ on NET release from neutrophils stimulated with PMA, HOCl or nigericin. Panels (**A**–**C**): Differentiated PLB-985 cells (1 × 10^6^ cells) were incubated with SCN^−^ (25–400 μM) for 15 min at 37 °C prior to the addition of (**A**) PMA (200 nM), (**B**) HOCl (0.75 mM) or (**C**) nigericin (15 μM) for 4 h at 37 °C. Panels (**D**–**F**): Primary neutrophils (1 × 10^6^ cells) were incubated with SCN^−^ (50–400 μM) for 15 min at 37 °C prior to the addition of (**D**) PMA (50 nM), (**E**) HOCl (0.75 mM) or (**F**) nigericin (15 μM) for 3 h at 37 °C. Following treatment, NETs were released by the addition of DNase (40 U) for 15 min and quantification of extracellular DNA using a Quant-iT^TM^ PicoGreen^TM^ dsDNA assay kit. Data are presented as mean ± S.E.M. from at least 3 experiments. *, **, *** and **** indicate a significant (*p* < 0.05, 0.01, 0.001, 0.0001) change compared to PMA-, HOCl- or nigericin-stimulated cells in the absence of SCN^−^ (white bars), while #, ##, ###, and #### indicate a significant (*p* < 0.05, 0.01, 0.001, 0.0001) change compared to untreated cells (black bars) as determined by one-way ANOVA with Dunnett’s multiple comparisons post hoc test.

**Figure 5:**
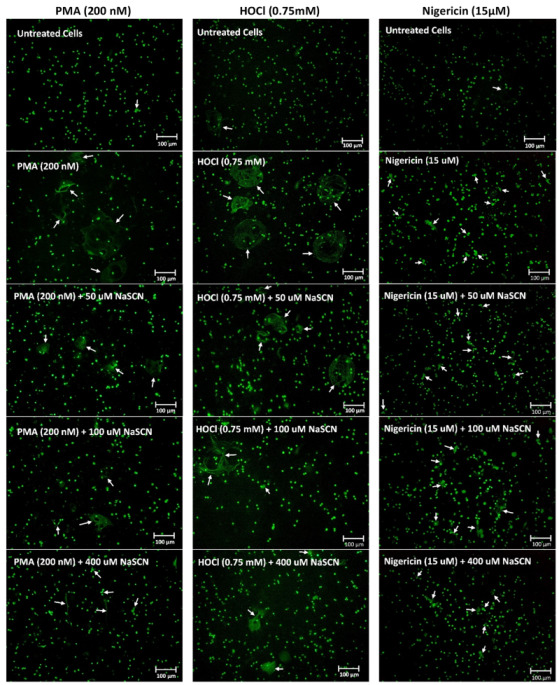
Microscopy of differentiated PLB-985 cells pre-treated with SCN^−^ prior to NET stimulation. Differentiated PLB-985 cells (4 × 10^5^) were treated with increasing concentrations of SCN^−^ for 15 min at 37 °C prior to the stimulation of cells with PMA (200 nM), HOCl (0.75 mM) or nigericin (15 μM) for 4 h. After the treatment, the cells were fixed in 4% formaldehyde for 30 min and the DNA was stained with SYTOX green (2 µM) for 10 min in the dark. The NETs are indicated by the white arrows. Images are representative of at least 3 independent experiments.

Exposure of PLB-985 cells to HOCl was shown to promote NET release, consistent with previous studies with primary neutrophils (reviewed [12]). However, HOCl production was only observed on exposure of the PLB-985 cells to PMA and not nigericin, though both treatments were shown to stimulate NET release. Therefore, different HOCl scavengers and/or MPO inhibitors were examined for their ability to modulate NET release in neutrophils stimulated with PMA, HOCl and nigericin. Initial studies were performed with SCN^−^, which can react directly with HOCl and act as a substrate for MPO [47,48]. The differentiated PLB-985 cells were pre-treated with SCN^−^ for 15 min before the addition of PMA (200 nM), HOCl (0.75 mM) or nigericin (15 µM) and extracellular DNA quantification with PicoGreen (Figure 4A–C) or visualization with SYTOX green and microscopy (Figure 5). A decrease in the release of extracellular DNA was observed on treatment of the PLB-985 cells with PMA or HOCl in the presence of increasing concentrations of SCN^−^ (50–400 µM). However, it is noted that with PMA, the increase in DNA release quantified by PicoGreen was not significant compared to the non-treated control (Figure 4A), though an increase in NET release was clearly seen by microscopy and SYTOX green under identical conditions (Figure 5). The reason for this is not certain but appears to be associated with a higher, background, fluorescence in the control, non-treated cells. In contrast, SCN^−^ had a less marked effect on the nigericin-stimulated release of extracellular DNA release from the PLB-985 cells (Figure 4C and Figure 5). These studies were extended to primary human neutrophils, where NET release was confirmed on exposure of the cells to PMA (50 nM), HOCl (0.75 mM) or nigericin (15 µM) by immunocytochemistry and colocalization of DNA with MPO and neutrophil elastase (Figures S4–S6). A similar pattern of reactivity was observed with the primary neutrophils, though a greater increase in PicoGreen fluorescence was seen compared to the control cells, consistent with a greater extent of extracellular DNA release (Figure 4D–F). However, with the primary neutrophils, there was significant variation in the magnitude of the PicoGreen fluorescence changes seen on stimulation of the cells from different cell donors. Therefore, with the primary neutrophils, the release of DNA has been expressed as a percentage of that observed with PMA, HOCl or nigericin in the absence of SCN^−^. In each case, SCN^−^ alone had no effect on DNA release compared to the non-treated cells (Figure 4D–F).

**Figure 6:**
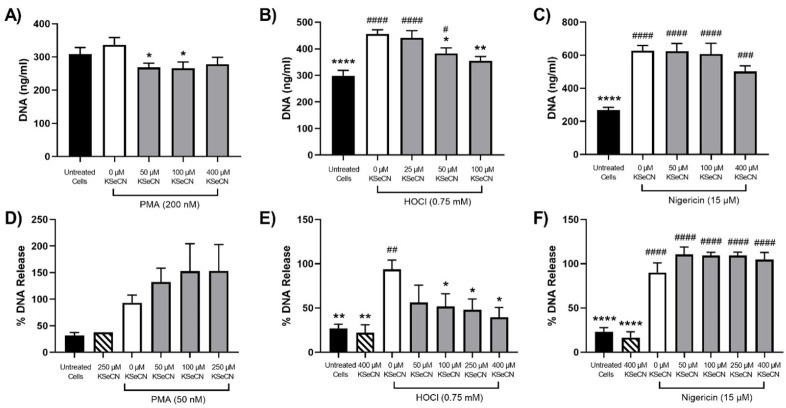
Effect of SeCN^−^ on NET release from neutrophils stimulated with PMA, HOCl or nigericin. Panels (**A**–**C**): differentiated PLB-985 cells (1 × 10^6^ cells) were incubated with SeCN^−^ (25–400 μM) for 15 min at 37 °C prior to the addition of (**A**) PMA (200 nM), (**B**) HOCl (0.75 mM) or (**C**) nigericin (15 μM) for 4 h at 37 °C. Panels (**D**–**F**): primary neutrophils (1 × 10^6^ cells) were incubated with SeCN^−^ (50–400 μM) for 15 min at 37 °C prior to the addition of (**D**) PMA (50 nM), (**E**) HOCl (0.75 mM) or (**F**) nigericin (15 μM) for 3 h at 37 °C. Following treatment, NETs were released by the addition of DNase (40 U) for 15 min and quantification of extracellular DNA using a Quant-iT^TM^ PicoGreen^TM^ dsDNA Assay kit. Data are presented and analysed as described in Figure 4.

Experiments were also performed using the selenium analogue SeCN^−^, which may be a more potent antioxidant owing to its more rapid reaction with HOCl [39]. SeCN^−^ (50–250 µM) decreased the release of NETs in a dose-dependent manner in PLB-985 cells stimulated with PMA (200 nM) and HOCl (0.75 mM) but not with nigericin (15 µM) (Figure 6A–C). Again, these data were confirmed by microscopy using SYTOX green (Figure S7). The inhibitory effect of SeCN^−^ was also examined in experiments with primary neutrophils (Figure 6D–F). With HOCl and nigericin, the effects of SeCN^−^ are comparable to the results seen with PLB-985 cells. However, with PMA, SeCN^−^ had the reverse effect, and was found to further stimulate the release of extracellular DNA (Figure 6D). The reason for this is not clear, as treatment of the neutrophils with SeCN^−^ (250 µM) alone did not result in the release of DNA (Figure 6D).

**Figure 7:**
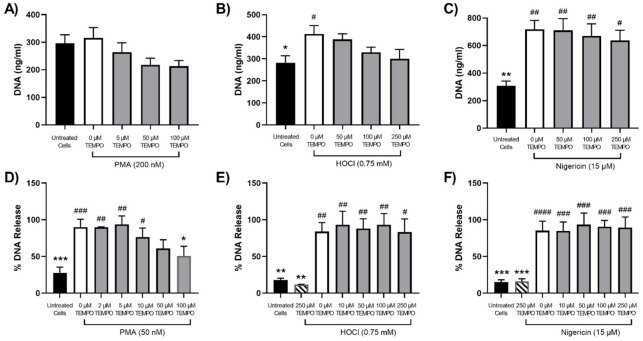
Effect of TEMPO on NET release from neutrophils stimulated with PMA, HOCl or nigericin. Panels (**A**–**C**): differentiated PLB-985 cells (1 × 10^6^ cells) were incubated with TEMPO (5–250 μM) for 15 min at 37 °C prior to the addition of (**A**) PMA (200 nM), (**B**) HOCl (0.75 mM) or (**C**) nigericin (15 μM) for 4 h at 37 °C. Panels (**D**–**F**): primary neutrophils (1 × 10^6^ cells) were incubated with TEMPO (2–250 μM) for 15 min at 37 °C prior to the addition of (**D**) PMA (50 nM), (**E**) HOCl (0.75 mM) or (**F**) nigericin (15 μM) for 3 h at 37 °C. Following treatment, NETs were released by the addition of DNase (40 U) for 15 min and quantification of extracellular DNA using a Quant-iT^TM^ PicoGreen^TM^ dsDNA Assay kit. Data are presented and analysed as described in Figure 4.

**Figure 8:**
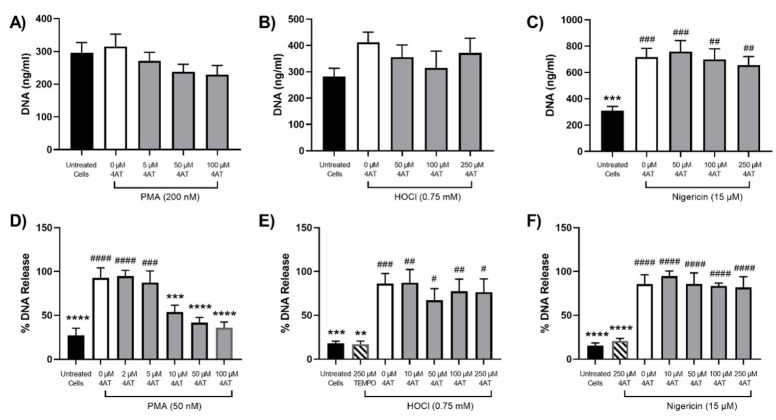
Effect of 4-amino TEMPO on NET release from neutrophils stimulated with PMA, HOCl or nigericin. Panels (**A**–**C**): differentiated PLB-985 cells (1 × 10^6^ cells) were incubated with 4-amino (5–250 μM) for 15 min at 37 °C prior to the addition of (**A**) PMA (200 nM), (**B**) HOCl (0.75 mM) or (**C**) nigericin (15 μM) for 4 h at 37 °C. Panels (**D**–**F**): primary neutrophils (1 × 10^6^ cells) were incubated with 4-amino TEMPO (2–250 μM) for 15 min at 37 °C prior to the addition of (**D**) PMA (50 nM), (**E**) HOCl (0.75 mM) or (**F**) nigericin (15 μM) for 3 h at 37 °C. Following treatment, NETs were released by the addition of DNase (40 U) for 15 min and quantification of extracellular DNA using a Quant-iT^TM^ PicoGreen^TM^ dsDNA Assay kit. Data are presented and analysed as described in Figure 4.

Although SCN^−^ and SeCN^−^ were shown to be effective at preventing NET release under some conditions, this approach would result in the production of other potentially reactive compounds [39,47,48]. Therefore, experiments were also performed with the nitroxides TEMPO and 4-amino TEMPO, which are known to inhibit MPO [38]. The differentiated PLB-985 cells and primary neutrophils were treated with TEMPO or 4-amino TEMPO for 15 min prior to stimulation with PMA, HOCl or nigericin, as outlined above. With the PLB-985 cells, TEMPO and 4-amino TEMPO (50–250 µM) had a slight inhibitory effect on NET release, but overall, none of the changes were statistically significant (Figure 7A and Figure 8A). However, with the primary neutrophils, a significant, dose-dependent, decrease in NET release was seen on stimulation with PMA (50 mM) in the presence of TEMPO and 4-amino TEMPO (2–100 µM) (Figure 7D and Figure 8D). A greater inhibitory effect on NET release was seen with 4-amino TEMPO compared to TEMPO (significance at 10 µM compared to 100 µM). In contrast, there was no significant inhibitory effect of TEMPO or 4-amino TEMPO on NET release in either PLB-985 cells or primary neutrophils on stimulation of the cells with HOCl or nigericin (Figure 7 and Figure 8).

## 4. Discussion

There is significant evidence for the involvement of NETs in the development of an increasing number of acute and chronic human diseases [6,20]. This has led to growing interest in the development of therapeutic approaches to target NETs, particularly those released because of prolonged exposure of neutrophils to pro-inflammatory, rather than pathogenic, stimuli [30,31]. MPO and HOCl are implicated in triggering NET release on exposure of neutrophils to different inflammatory stimuli [6,12]. Therefore, in this study, we examined the efficacy of different antioxidants known to scavenge HOCl and/or modulate MPO activity to prevent NET release from PLB-985 cells and primary human neutrophils. We showed that PLB-985 cells differentiated with ATRA and DMSO could release NETs upon treatment with PMA, HOCl or nigericin, but that only stimulation with PMA, rather than nigericin, resulted in the production of HOCl. A decrease in NET release to varying extents was observed in the treatment of the neutrophils with PMA in the presence of SCN^−^, SeCN^−^, TEMPO or 4-amino-TEMPO. However, these compounds were unable to prevent the NET release triggered by nigericin.

The PLB-985 cell line is a sub-clone of the HL-60 cell line [49] and can be differentiated into mature, neutrophil-like cells using different compounds, including dimethyl formamide (DMF), DMSO and ATRA [50,51,52]. ATRA is a metabolite from vitamin A shown to have chemotherapeutic effects on various cancer cells [53]. It can induce cell cycle arrest and terminal neutrophil differentiation, with a combination of ATRA with DMSO reported to synergize this effect [50,52]. The chemical-induced differentiation mimics granulopoiesis, which is the usual maturation process where immature myeloblasts are converted to mature polymorphonuclear neutrophils [54]. The PLB-985 cell line has been used previously as a model of NET release (e.g., [55]). NET release was observed on the addition of PMA (10–250 µM) to PLB-985 cells differentiated 6 days with ATRA (2 µM) and DMF (0.5%). The NETs contained MPO and were able to effectively trap and kill bacteria, consistent with the production of HOCl [55].

Our data support this observation and provide the first direct evidence for the formation of HOCl in PLB-985 cells stimulated with PMA, but not nigericin. PMA promotes the influx of Ca^2+^ via the activation of PKC, which triggers the assembly of NADPH oxidase and the formation of O_2_^∙−^ [6,11]. Thus, stimulation of PLB-985 cells differentiated with DMSO (1.3%, 3 days) released O_2_^∙−^ upon treatment with PMA for 10 min, an effect that was enhanced by priming the cells with interferon-γ (IFN-γ), which upregulated the expression of the NADPH oxidase 2 proteins gp91phox, p47phox and p22phox [56]. In contrast, nigericin is a K^+^ ionophore, which promotes the influx of Ca^2+^ by inducing the efflux of mitochondrial K^+^ [57]. This induces NET release independently of PKC and NADPH oxidase activation [11]. However, it is possible that nigericin may also induce some NET release via an NADPH oxidase-dependent pathway, owing to the potential activation of the NLR family, pyrin domain-containing 3 (NLRP3) inflammasome as a result of the influx of K^+^ [57]. Inflammasome activation would induce the release of interleukin 1β (IL-1β), which could then trigger the release of NETs from other neutrophils in a similar manner to PMA [9].

In addition to PKC, Ca^2+^ and O_2_^∙−^, MPO and HOCl are also involved in NETosis induced by PMA, and inhibition of MPO can block this process [11,12]. Pre-treatment of the differentiated PLB-985 cells with SCN^−^ or SeCN^−^ prior to PMA treatment resulted in a decrease in NET release, which was not seen in the nigericin-treated cells. These data are consistent with the known differences in the pathways responsible for NET release and are supported by previous studies with the MPO inhibitor 4-aminobenzoic acid hydrazide (ABAH) [11]. SCN^−^ and SeCN^−^ were also able to prevent NET release induced by exposure of the PLB-985 cells to HOCl. Taken together, these observations are attributed to the ability of these compounds to act as both alternative substrates for MPO and direct scavengers of HOCl [39,58]. This results in the formation of the alternative oxidants, hypothiocyanous acid (HOSCN) and hyposelenocyanous acid (HOSeCN). HOSCN is a milder oxidant than HOCl and reacts selectively with thiols to form mainly reversible oxidation products [47,48]. As such, there is evidence that HOSCN-induced damage can be repaired [39,59], though this oxidant is cytotoxic at high concentrations or on prolonged incubation [47,48]. Less is known about the reactivity of HOSeCN, but recent studies suggest that it is less toxic than HOCl to mammalian cells [39,60]. Analogous results were obtained with primary human neutrophils, with the exception that SeCN^−^ appeared to increase NET release in PMA-stimulated cells. The reason for this is not certain, as treatment of the neutrophils with SeCN^−^ in the absence of PMA had no apparent toxicity, and protection was seen in experiments with HOCl. Previous studies with selenomethionine (SeMet), which also readily scavenges HOCl [61], demonstrated a decrease in NET release from primary neutrophils treated with PMA [36]. Similarly, a deficiency in selenium has been linked to increased NET release ex vivo and in the aortae of chicken [62]. It is also possible that the preparations of human neutrophils used may contain some residual monocytes, which could also potentially contribute to the release of extracellular traps. Structurally, the extracellular traps from monocytes are reported to be similar to neutrophils, but there may be differences in the mechanisms triggering trap release [63].

Further studies will be needed to examine the influence of SCN^−^ and SeCN^−^ on NET release from PMA and other pro-inflammatory stimuli in more detail, as this supplementation approach could be advantageous in chronic inflammatory conditions. Thus, the addition of SCN^−^ or SeCN^−^ will result in a decrease in HOCl and a corresponding increase in the formation of HOSCN or HOSeCN, which are anti-microbial, but postulated to cause less damage to host cells [47,60,64]. HOSeCN is reported to kill bacteria to a similar extent to HOCl and even more potently than HOSCN [60]. In addition, mammalian, but not bacterial, thioredoxin reductase (TrxR) enzymes can remove HOSeCN and HOSCN, providing further support that these oxidants will be less damaging to host cells [60,65]. This could overcome the limitation of long-term MPO inhibitors on innate immunity and clearing infection, and provides a possible additional benefit, as SeCN^−^ can be metabolised by cells and incorporated into selenoproteins [66], which have potent antioxidant abilities and improve cell survival [67].

The nitroxides TEMPO and 4-amino-TEMPO were effective at decreasing NET release from primary neutrophils stimulated with PMA, but not HOCl or nigericin. This is attributed to the ability of these compounds to remove O_2_^∙−^ produced by NADPH oxidase, and inhibit MPO, rather than by reacting with HOCl directly [38,68]. Which mechanism of inhibition is more dominant is not certain. 4-amino-TEMPO is the more potent MPO inhibitor and gave a more significant decrease in NET release compared to TEMPO. However, 4-amino-TEMPO also has potent SOD-mimetic activity [38]. As with the selenium compounds, there was no effect of TEMPO or 4-amino-TEMPO on nigericin-induced NET release in either cell type, consistent with the NADPH-independent pathway of NETosis [11]. Previous studies have shown that 4-hydroxy-TEMPO (Tempol) inhibits NET release in neutrophils treated with PMA and *Candida albicans*, consistent with our study [69]. However, although a dose-dependent effect of Tempol on NET release was apparent, much higher concentrations of the nitroxide were employed (10–30 mM), compared to those used here. Thus, TEMPO and 4-amino-TEMPO were effective at much lower concentrations (10–100 µM). Interestingly, other studies have shown that the mitochondrially-targeted nitroxide, mito-TEMPO, can decrease NET release from differentiated HL-60 cells stimulated with the Ca^2+^ ionophore A23187 but not PMA [70]. This is attributed to the role of mitochondrial ROS in NET release triggered by A23187, rather than by activation of NADPH oxidase, as seen with PMA [70]. This raises the possibility of selectively modulating NET release from different inducers using either targeted or non-targeted nitroxides. A limitation of our study is that the efficacy of mito-TEMPO was not examined, as this might have been effective with nigericin, based on the suggestion of a role for mitochondrial ROS with this inducer, analogous to A23187 [11,70].

There are challenges associated with selectively targeting only pathological NET release, given that there are similarities in the mechanisms of inflammatory and pathogen-induced NETosis [6,11]. Prolonged inhibition of NET release could render individuals more susceptible to infections, though it is possible with some therapeutic approaches to leave other neutrophil-killing mechanisms intact. A popular approach has been to selectively inhibit the release of NETs, while allowing other protective neutrophil actions, by inhibition of peptidylarginine deiminase (PAD4) with Cl-amidine [71,72] or GSK199 and GSK484 [31]. The activation of PAD4 converts the arginine residues of histones into citrulline, which promotes chromatin unfolding to stimulate the release of NETs [6]. However, while citrullinated histones are widely used as markers for NET release [30], citrullination is not always a requirement for NET release [15,16], particularly in chronic conditions such as atherosclerosis [73,74]. Similarly, degradation of the released NETs with DNase, while effective at removing the DNA [31,75], could result in the accumulation of NET components, which may cause undesirable effects [76].

## 5. Conclusions

In summary, our data provide support for the possibility of selectively regulating NETosis with different inducers. The use of SCN^−^ and SeCN^−^ could result in a decrease in NET release and HOCl production to minimise host cell damage, while still allowing bacterial killing. The nitroxides TEMPO and 4-amino-TEMPO are effective at decreasing NET release induced by PMA as a model inflammatory stimulus at low concentrations (≤100 µM). These concentrations of nitroxide are non-toxic and would be readily achievable in vivo [77]. Further studies will be required to assess the utility of these approaches in a pathological context, owing to the multiple actions of these compounds. However, their ability to exert other anti-inflammatory and/or radical scavenging effects could ultimately also be advantageous, particularly in chronic inflammatory disease, where there is an infiltration of neutrophils and other immune cells and activation of many damaging pathways.

## Data Availability

Data is contained within the article and supplementary material.

## Supplementary Materials

The following supporting information can be downloaded at: https://www.mdpi.com/article/10.3390/antiox12020478/s1, Figure S1: PLB-985 cells differentiate upon treatment with ATRA and DMSO; Figure S2: Expression of CD11b on differentiation of PLB-985 cells with ATRA and DMSO; Figure S3: Colocalization of extracellular DNA and citrullinated histone H3 in differentiated PLB-985 cells exposed to PMA; Figure S4: Immunocytochemistry showing the presence of MPO and elastase on NETs released by neutrophils stimulated by PMA; Figure S5: Immunocytochemistry showing the presence of MPO and elastase on NETs released by neutrophils stimulated by HOCl; Figure S6: Immunocytochemistry showing the presence of MPO and elastase on NETs released by neutrophils stimulated by nigericin; Figure S7: Microscopy of differentiated PLB-985 cells pre-treated with SeCN^−^ prior to NET stimulation.
