# Supplementary material for: Antioxidant Strategies to Modulate NETosis and the Release of Neutrophil Extracellular Traps during Chronic Inflammation
Source: Antioxidants (Basel). 2023 Feb 14;12(2):478. doi: 10.3390/antiox12020478 (PMC9952818; doi:10.3390/antiox12020478)
Supplement: Supplementary file 1 [file antioxidants-12-00478-s001.zip › antioxidants-2221909-supplementary.pdf]

SUPPLEMENTARY DATA

Antioxidant Strategies to Modulate NETosis and the Release of Neutrophil Extracellular Traps During Chronic Inflammation

Line A.E. Hallberg, Kristine Barlous and Clare L. Hawkins*

Department of Biomedical Sciences, University of Copenhagen, DK-2200 Copenhagen, Denmark

---

*Correspondence: clare.hawkins@sund.ku.dk

Supplementary Figure S1

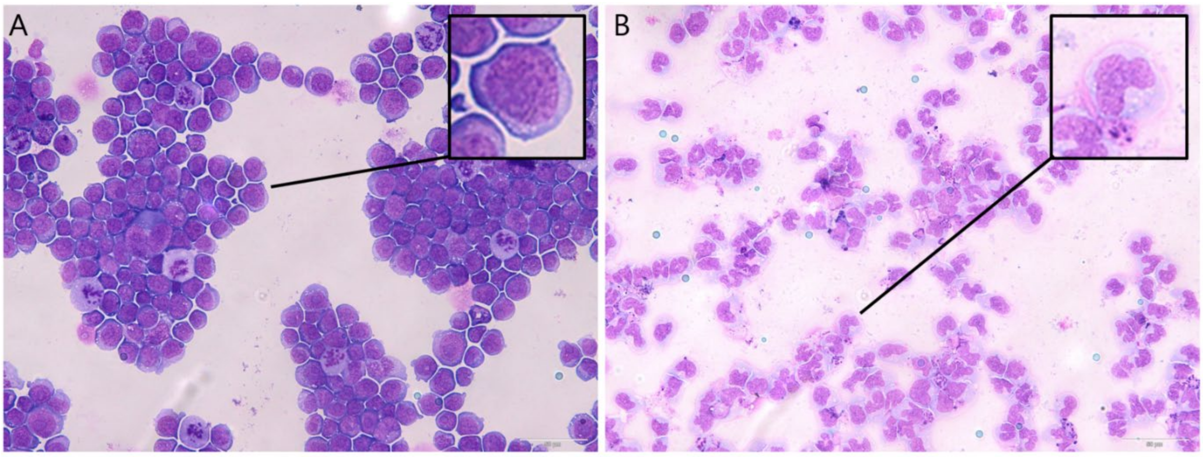

Figure S1: PLB-985 cells differentiate upon treatment with ATRA and DMSO.

PLB-985 cells (0.3 x 10^6 cells/mL) were treated with ATRA (2 µM) and DMSO (1.3%) for either 0 h (A) or 72 h (B) before May-Grünwald-Giemsa staining. Upper right corner of each image shows an increased magnification of a single cell. Scale bar shows 50 µM. Images are representative of 3 independent experiments.

Supplementary Figure S2

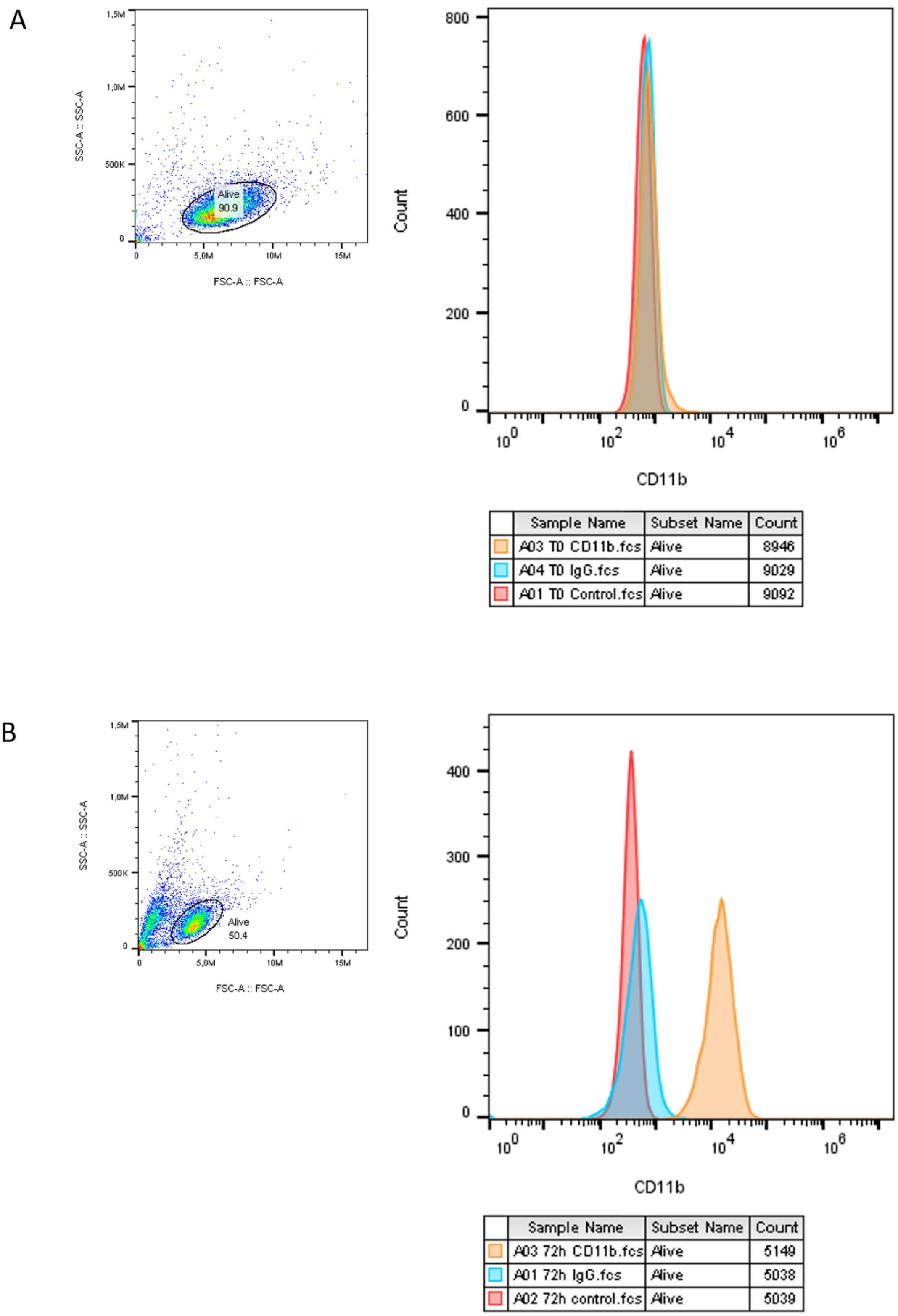

Figure S2: Expression of CD11b on differentiation of PLB-985 cells with ATRA and DMSO.

PLB-985 cells (0.3 x 10^6 cells/mL) were differentiated with ATRA (2 µM) and DMSO (1.3%) for either 0 h (A) or 72 h (B) before analysis of the expression of CD11b, a neutrophil maturation marker, using flow cytometry. The control is indicated in red, the IgG isotype control is indicated in blue and the CD11b positive cells are shown in yellow. Dead cells and cell debris were excluded by staining with 7-aminoactinomycin D.

Supplementary Figure S3

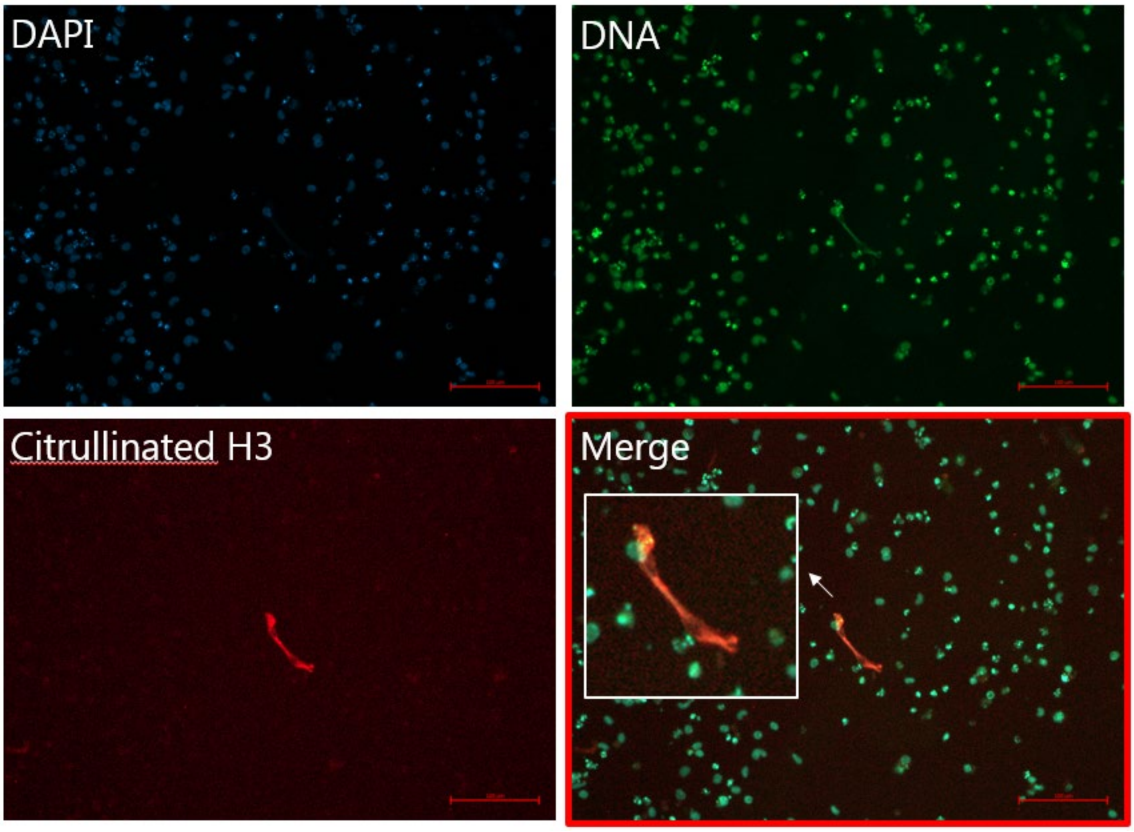

Figure S3: Colocalization of extracellular DNA and citrullinated histone H3 in differentiated PLB-985 cells exposed to PMA.

PLB-985 cells (4 x 10^5 cells) differentiated with ATRA (2 µM) and DMSO (1.3%) for 72 h were treated with PMA (50 nM) for 4 h before fixing with 4% formaldehyde for 20 min and washing in PBST. To avoid unspecific binding, the cells were incubated with serum blocker (5% BSA in PBST) overnight at 4 °C, followed by incubation with an anti-histone citrulline H3 antibody for 2 h at 21°C. Cells were washed in PBST and incubated with a secondary antibody (AlexaFluor® 647) for 2 h in the dark. Cells were washed in PBST before incubation with SYTOX green (2 µM) and DAPI (1:1000) for 15 min. The slides were imaged on a ZEISS widefield fluorescent microscope. Blue: DNA stained with DAPI. Green: DNA stained with SYTOX green. Red: citrullinated histone 3. The image highlighted with a red border shows the merged image. Scale bar 100 µm.

Supplementary Figure S4

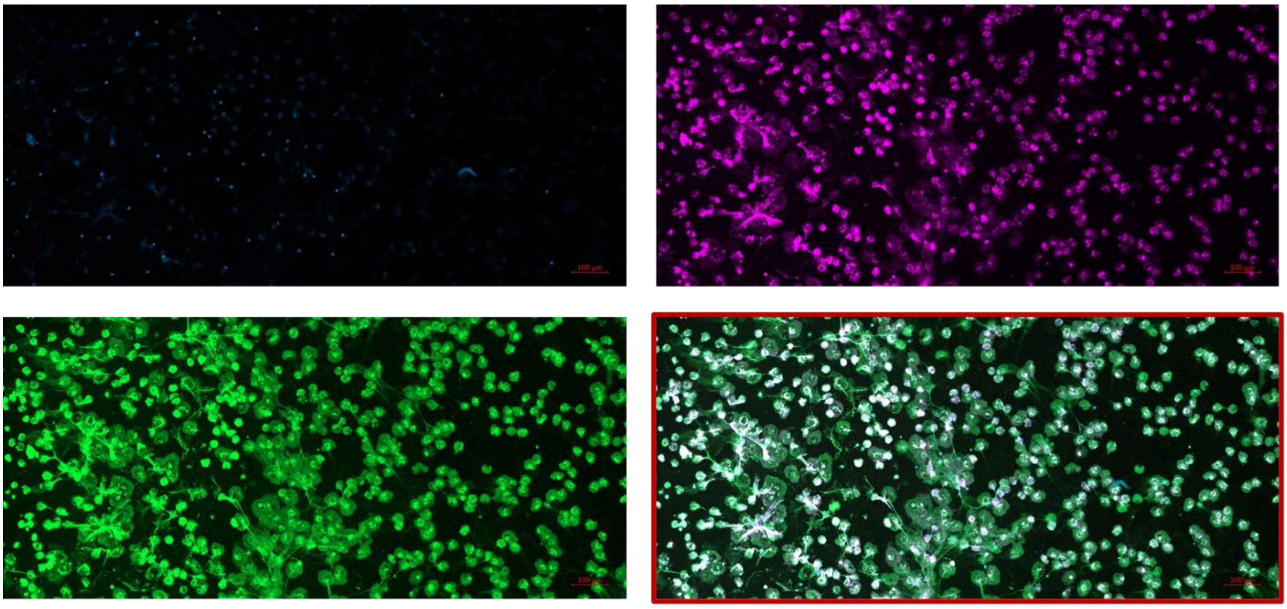

Figure S4: Immunocytochemistry showing the presence of MPO and elastase on NETs released by neutrophils stimulated by PMA.

Primary neutrophils (0.5 x 10^6 cells/ml) were treated with PMA (50 nM) for 3 h at 37 °C. The cells were fixed with 4% formaldehyde for 30 min and washed in PBST. To avoid unspecific binding, the cells were incubated with serum blocker (5% BSA in PBST) overnight at 4 °C, followed by incubation with primary antibodies (anti-MPO ab25989 mouse and anti-elastase ab21595 rabbit) for 2 h at 21 °C. Cells were washed in PBST and incubated with secondary antibodies for 2 h in the dark (AlexaFluor® 488 for elastase and AlexaFluor® 647 for MPO). Cells were then washed in PBST before incubation with DAPI (1:1000) for 15 min and imaging on a fluorescent microscope (ZEISS Axio Scan.Z1). Blue: DAPI. Pink: MPO. Green: Elastase. Red highlighted: merge. Scale bar 100 µm.

Supplementary Figure S5

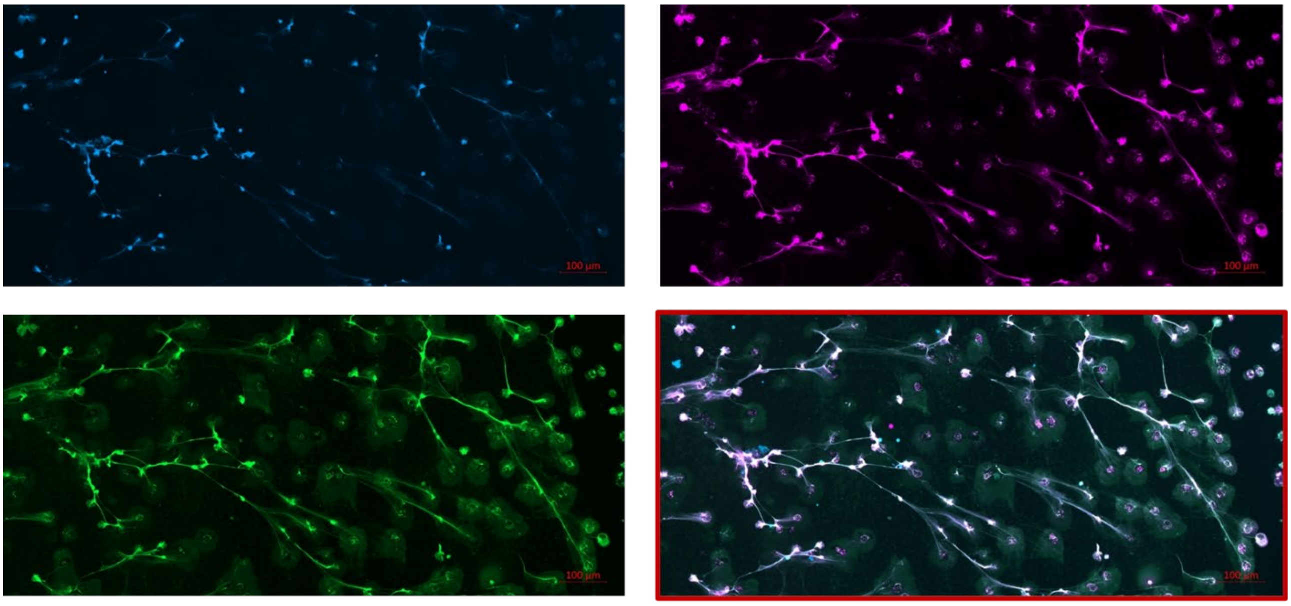

Figure S5: Immunocytochemistry showing the presence of MPO and elastase on NETs released by neutrophils stimulated by HOCl.

Primary neutrophils (0.5 x 10^6 cells/ml) were treated with HOCl (0.75 mM) for 3 h at 37 °C. The cells were fixed with 4% formaldehyde for 30 min and washed in PBST. To avoid unspecific binding, the cells were incubated with serum blocker (5% BSA in PBST) overnight at 4 °C, followed by incubation with primary antibodies (anti-MPO ab25989 mouse and anti-elastase ab21595 rabbit) for 2 h at 21 °C. Cells were washed in PBST and incubated with secondary antibodies for 2 h in the dark (AlexaFluor® 488 for elastase and AlexaFluor® 647 for MPO). Cells were then washed in PBST before incubation with DAPI (1:1000) for 15 min and imaging on a fluorescent microscope (ZEISS Axio Scan.Z1). Blue: DAPI. Pink: MPO. Green: Elastase. Red highlighted: merge. Scale bar 100 µm.

Supplementary Figure S6

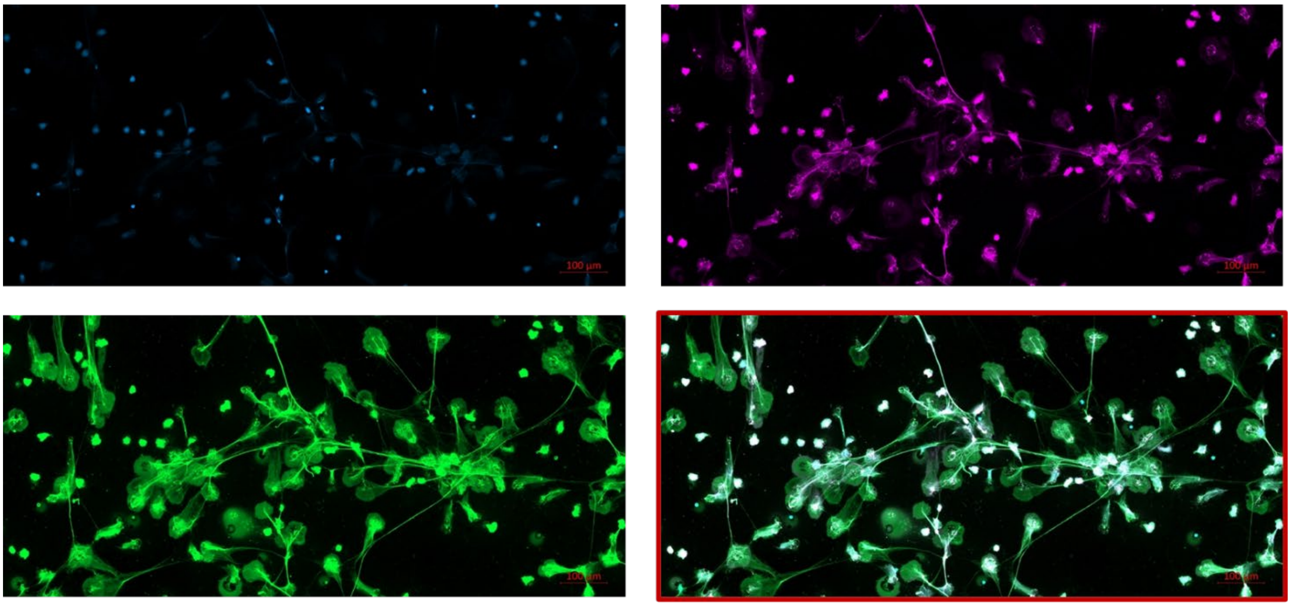

Figure S6: Immunocytochemistry showing the presence of MPO and elastase on NETs released by neutrophils stimulated by nigericin.

Primary neutrophils (0.5 x 10^6 cells/ml) were treated with nigericin (15 µM) for 3 h at 37 °C. The cells were fixed with 4% formaldehyde for 30 min and washed in PBST. To avoid unspecific binding, the cells were incubated with serum blocker (5% BSA in PBST) overnight at 4 °C, followed by incubation with primary antibodies (anti-MPO ab25989 mouse and anti-elastase ab21595 rabbit) for 2 h at 21 °C. Cells were washed in PBST and incubated with secondary antibodies for 2 h in the dark (AlexaFluor® 488 for elastase and AlexaFluor® 647 for MPO). Cells were then washed in PBST before incubation with DAPI (1:1000) for 15 min and imaging on a fluorescent microscope (ZEISS Axio Scan.Z1). Blue: DAPI. Pink: MPO. Green: Elastase. Red highlighted: merge. Scale bar 100 µm.

Supplementary Figure S7

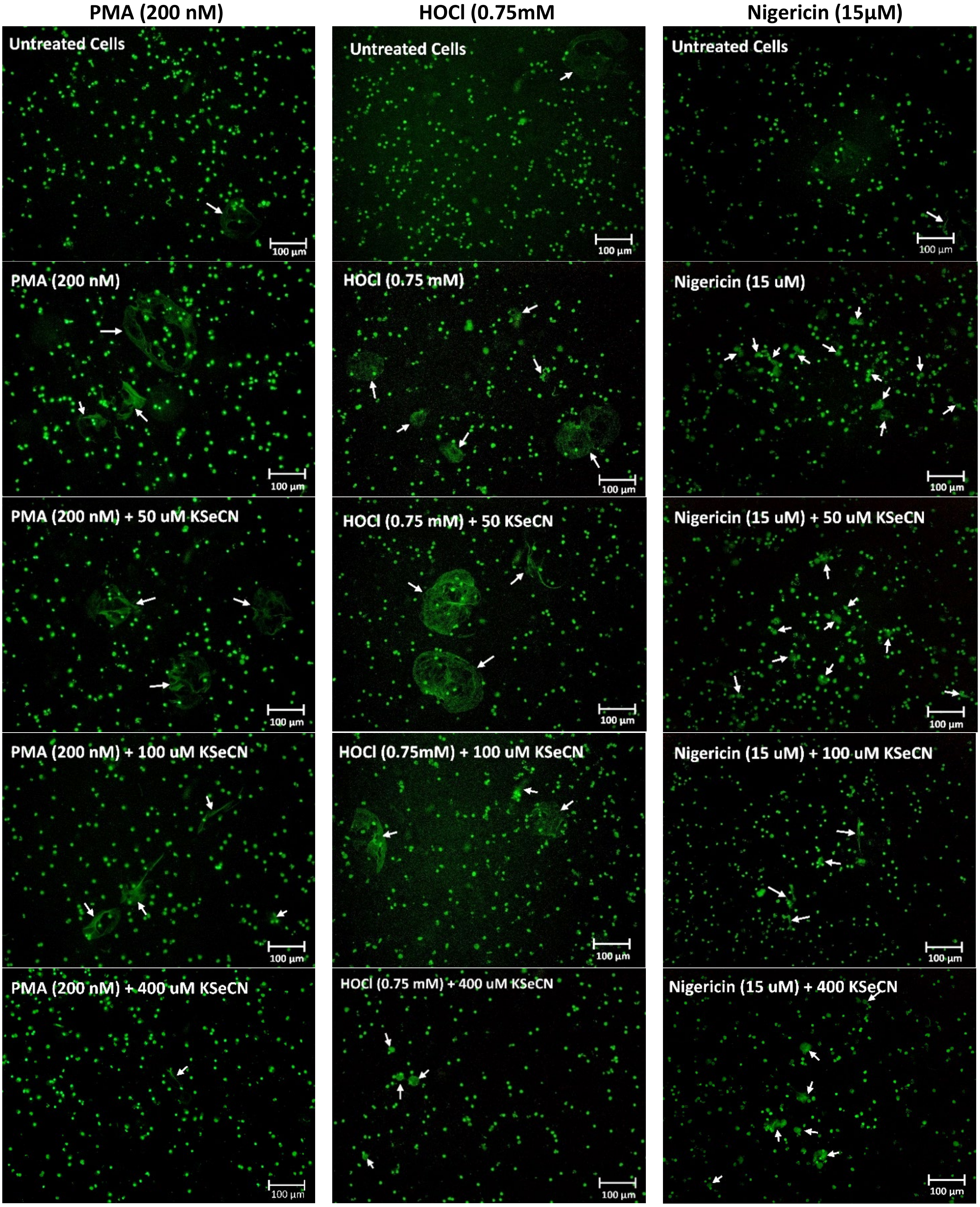

Figure S7: Microscopy of differentiated PLB-985 cells pre-treated with $SeCN^-$ prior to NET stimulation. Differentiated PLB-985 cells (4 x 10^5 cells) were treated with $SeCN^-$ (50 – 400 µM) for 15 min at 37 °C prior to stimulation of cells with PMA (200 nM), HOCl (0.75 mM) or nigericin (15 µM) for 4 h. After treatment, cells were fixed in 4% formaldehyde for 30 min and DNA was stained with SYTOX green (2 µM) for 10 min. The NETs are indicated by the white arrows. Images are representative of at least 3 independent experiments.
